# A Novel Flow Chemistry Approach to Covalent Functionalization of 3D Graphene Aerogels

**DOI:** 10.1021/acsomega.5c02481

**Published:** 2025-07-07

**Authors:** Antonino Biagio Carbonaro, Valentina Greco, Valentina Pifferi, Luigi Falciola, Enrico Ciliberto, Antonino Gulino, Alessandro Giuffrida

**Affiliations:** † Department of Chemical Sciences, 9298 Università degli Studi di Catania, Viale A. Doria 6, 95100 Catania, Italy; ‡ Department of Chemistry, 9304 Università degli Studi di Milano, via Golgi 19, 20133 Milano, Italy; § Consorzio Interuniversitario Nazionale per la Scienza e Tecnologia dei Materiali (INSTM), Via G. Giusti 9, 50121 Firenze, Italy

## Abstract

Tuning the surface chemistry of 3D graphene structures, such as hydrogels and aerogels, is critical for advancing their chemical and physical properties, which are essential for material design. Here, we present an innovative in-flow covalent functionalization approach based on diazonium salt chemistry to introduce new functionalities into the 3D graphene aerogel backbone while preserving its porous architecture. To achieve this, we designed a flow-based reactor tailored for the functionalization of macroscopic aerogel samples, addressing limitations of noncovalent methods including molecular slippage. Notably, the proposed method operates at room temperature, a significant advantage over existing techniques that often require high thermal conditions. Additionally, to overcome challenges associated with solid-state Raman analysis of graphene-based compounds, we proposed a statistical model to enhance the reproducibility of the process and rationalize *I*_D_/*I*_G_ ratios post-treatment. This work demonstrates the feasibility of in-flow covalent functionalization of 3D graphene aerogels, opening new perspectives for the development of customizable porous carbon-based materials for various technological applications.

## Graphical abstract

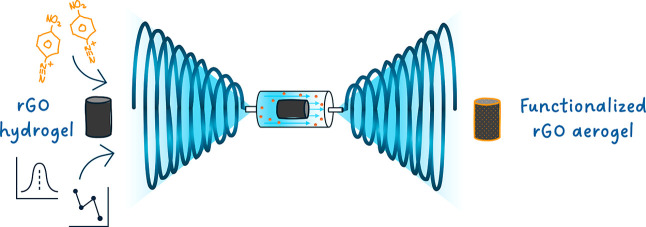

## Introduction

Over the past decade, porous graphene aerogels have gained significant attention from the scientific community due to their unique structural, chemical, and physical properties.^1^ When combined with the extensively studied 2D graphene, these aerogels have demonstrated potential across various applications, including bioelectronics,^2,3^ sensing,^4–6^ pollution remediation,^7^ electromagnetic interference shielding materials,^8^ and energy storage.^9^

In this context, 3D graphene structures, such as hydrogels and aerogels, represent a promising solution to enhance the properties of 2D graphene nanocomposites. The interconnected porous network of 3D graphene, combined with its inherent properties, provides an increased surface area and enhanced pathways for electron transport and storage. Consequently, 3D graphene exhibits a well-defined architecture, excellent electrical conductivity, a high specific surface area, and versatile gas adsorption sites.^10–13^

The interaction between the porous carbon matrix and a secondary phase, whether liquid or gaseous, is a critical factor for most applications of these materials. As a result, functionalizing graphene aerogels to introduce heteroatoms is essential to tailor their properties for specific requirements.^14^

The chemical modification of 3D graphene structures has emerged as a growing area of interest with the aim of optimizing these materials for targeted applications. For example, in the field of engineered living materials (ELMs), the ability to control the surface chemistry of graphene-based 3D scaffolds has become increasingly important. Modifying surface chemistry, alongside fine-tuning physicochemical properties, can significantly enhance parameters such as biocompatibility and reduce nanotoxicity.^15,16^ Furthermore, as highlighted by the work of Plata-Gryl et al., the surface chemistry of reduced graphene oxide aerogels plays a crucial role in their properties and applications, influencing factors such as surface free energy, roughness, and specific molecular interactions.^17^

Functionalization strategies for graphene-based materials generally depend on the surface chemistry of graphene oxide (GO), which is rich in oxygenated functional groups such as carboxyls, carbonyls, and hydroxyls, a powerful source for the material design.^18–21^ However, for the synthesis of graphene hydrogels and aerogels, GO must undergo reduction to induce spontaneous self-assembly, a process that removes most of these functional groups to form the reduced graphene oxide (rGO) 3D architecture. While the resulting 3D-rGO structure exhibits high surface area and pronounced electrical properties, the absence of functional groups renders these materials relatively inert, lacking catalytic or molecular recognition sites, which limits their functional versatility. In this context, the rational functionalization and the surface engineering of carbon-only graphene materials with active heteroatoms or guest components to imbue more appealing properties and practical applications become highly welcomed.^17,22^ Graphene aerogels are primarily functionalized by incorporating guest species into the colloidal GO dispersion prior to self-assembly, forming hybrid 3D structures with guest species embedded between rGO layers.^23–28^ However, noncovalent functionalization has limitations, such as molecular slippage due to weak interactions,^29^ and the need to ensure compatibility between guest species and the GO colloid to avoid chemical alterations during assembly. Experimental conditions, including the temperature, pressure, pH, and reducing agents, can significantly influence guest species. Alternatively, doping with heteroatoms (e.g., N, S, P, B) can alter graphene’s inertness but often requires high-temperature treatments, increasing costs for large-scale applications.^30–33^

To sum up, despite the fact that the modification of the surface chemistry of two-dimensional graphene has been extensively explored in the literature, with numerous synthetic strategies developed to control its chemical and physical properties, there is a lack of systematic studies on the modification of 3D graphene structures. A possible reason could be that, in the case of three-dimensional graphene structures like hydrogels and aerogels, classical bulk functionalization techniques, which require stirring to homogenize the reaction mixture, are less feasible due to the risk of compromising the material’s brittle porous microstructure.

Given the growing demand for three-dimensional graphene systems and the current difficulties and limitations in controlling the surface chemistry, in this work, we propose a covalent functionalization strategy to modify the porous structure of preformed graphene hydrogels by the well-established diazonium salt chemistry.^34,35^

Carbon skeleton grafting reactions are of great help in modifying a low-reactive material such as graphene in its functional group-free form, and, due to the versatility of aryl diazonium chemistry, diazonium salts are important building stones to construct complex interfaces covalently anchored to carbon surfaces.^36^ In this scenario, this functionalization approach is highly versatile for the introduction of new functional groups into graphene and its derivatives. In general, diazonium salt chemistry is employed either to tune the electronic properties or to anchor more complex organic molecules through the successive chemical reactions of the modified material.^35,37^ A variety of commercial aromatic amines are available with versatile functional groups suitable for successive reactions. Examples include 4-aminophenol (−COOH), 4-aminothiophenol (–SH), and 4-aniline-sulfonic acid (−SO_3_H).^38,39^ For example, Rebuttini et al. employed 4-aminophenol as the primary aromatic amine for spontaneous graphene oxide grafting, with the objective of investigating the interaction between iron oxide nanoparticles and the –COOH functional groups.^39^ Another example is represented by the work of Wang et al., in which Ar-NO_2_ moieties were grafted on rGO layers in order to enhance the adhesion of Prussian Blue structures for the development of sensors aimed at hydrogen peroxide detection.^40^ This strategy therefore offers broad opportunities for tailoring the surface chemistry of graphene and its derivatives. However, while graphene in its two-dimensional form is compatible with the experimental conditions of this reaction—since it can be readily dispersed in solution as a powder—this is not technically feasible for self-assembled structures such as graphene hydrogels and aerogels as their architecture would be compromised.

To address this challenge, we developed a custom-designed flow reactor that allowed us to effectively functionalize graphene hydrogel samples without compromising the porous structure, which were subsequently converted to functionalized graphene aerogels by freeze-drying. In this way, the reaction mixture can flow uniformly throughout the entire hydrogel structure without the need to disperse the material in a destructive manner. This method demonstrates a significant advantage, as it operates at room temperature and allows the use of small quantities of reagents for a single functionalization event.

To the best of our knowledge, this represents the first instance of covalent functionalization of preformed graphene aerogels by using a flow reactor. Additionally, inspired by the work of Wróblewska et al.,^41^ we implemented a statistical approach to process Raman spectroscopy data, ensuring the reproducibility of the functionalized graphene 3D structures. This model addresses a well-known limitation in the literature, wherein single Raman measurements are insufficient to accurately characterize samples that are chemically and morphologically heterogeneous.^42^

## Materials and Methods

### Chemicals

The graphene oxide water dispersion (0.4 wt % concentration, pH 2.2–2.5, monolayer content >95%) was purchased from Graphenea. l-Ascorbic acid (ACS reagent), HCl (37%, ACS reagent), NaNO_2_ (ACS reagent, ≥97.0%), absolute ethanol (≥99.5%, ACS reagent), and 4-nitroaniline (≥99%) were purchased from Sigma-Aldrich. All the chemicals were used as received. For all steps, Milli-Q water was used.

### Reactor Building

The experimental work was carried out using a home-built reactor, which was useful both during the removal of byproducts from the hydrogel and during the functionalization step. Figure 1a shows a schematic representation of the home-built reactor. All flow lines were made of PE, compatible with all of the reagents used during the experimental steps. The pneumatic pump module (1) operates with a N_2_ flow directed toward the solvent vessels (2). The nitrogen flow controls the flow of the solvent to the reaction chamber. It is also possible to switch the solvent vessel at this stage. The reaction mixture injector (3) is made of glass and through the switching valve (4) and allows a known amount of the reagent to be injected into the reaction chamber. The switching valve (4) allows the solvent line to be closed to open the reaction mixture line coming from the injector and allows the respective flows to be closed and opened to control the reaction time. In addition, by appropriately adjusting the position of the switching valve, it is possible to set the reactor in “washing mode”, a step that allows the sample to be rinsed for the removal of byproducts, conveying the wash water to the waste (7). The reaction chamber (5) is made of glass and has a cylindrical shape and a volume of 4 mL. The peristaltic pump module (6) is used to circulate the reaction mixture through the reaction chamber (Figure 1b). This allows the reaction mixture to diffuse through the hydrogel sample to achieve homogeneous surface functionalization of the 3D graphene structure.

**Figure 1:**
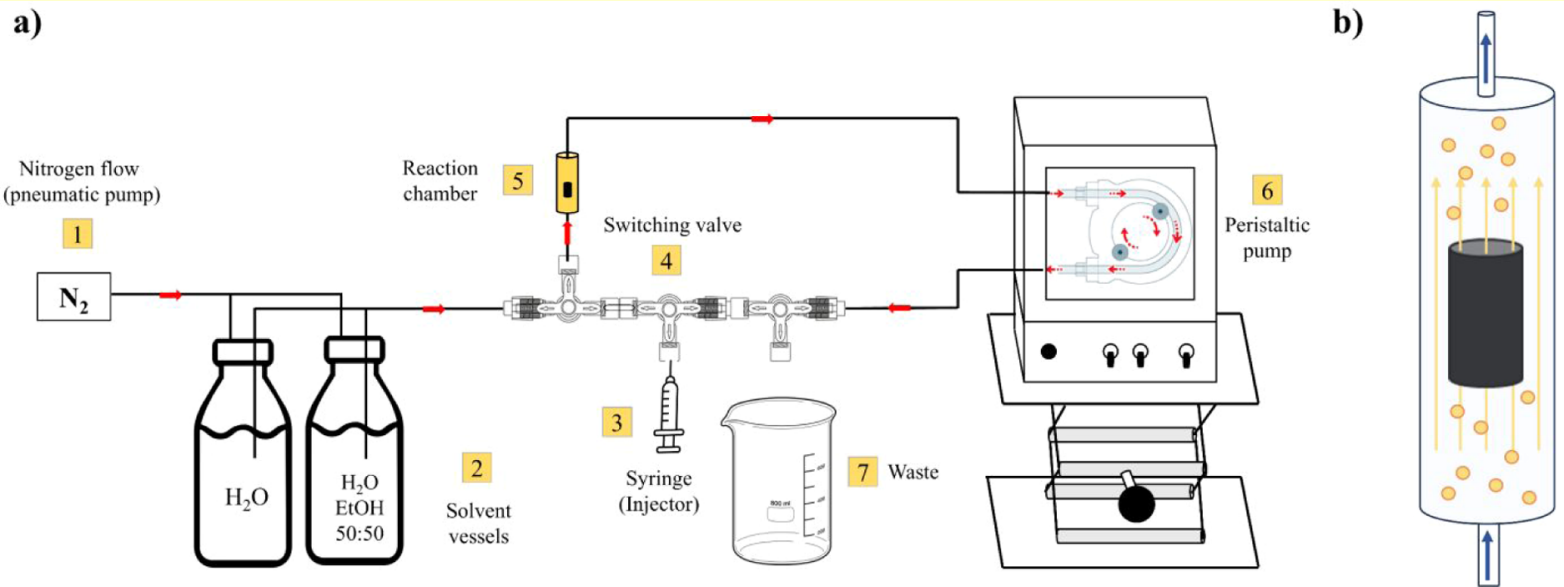
(a) Schematic representation of the flow reactor; (b) magnification of the reaction chamber showing the graphene hydrogel functionalization step; yellow dots represent the reaction mixture flowing from the bottom to the top of the reaction chamber.

### Synthesis of the Graphene Aerogel

The graphene hydrogel was prepared by a chemical reduction method using l-ascorbic acid as the reducing agent.^43–45^ Briefly, 750 μL of the GO water dispersion (3 mg/mL) was mixed with 750 μL of l-ascorbic acid (82 mM) into a glass test tube and, after stirring for 1 min, the mixture was placed into a preheated oven at 95 °C. During the reduction, the GO mixture turned from brown to black, and after 30 min, the rGO hydrogel structure was obtained (Figure 2a,b). The resulting hydrogel was placed inside the reaction chamber and was washed with excess l-ascorbic acid with a water flow (5 mL/min) for 2 h. Then, the clean hydrogel was freeze-dried (−80 °C, overnight) to form the rGO aerogel (rGOA) (Figure 2c).

**Figure 2:**
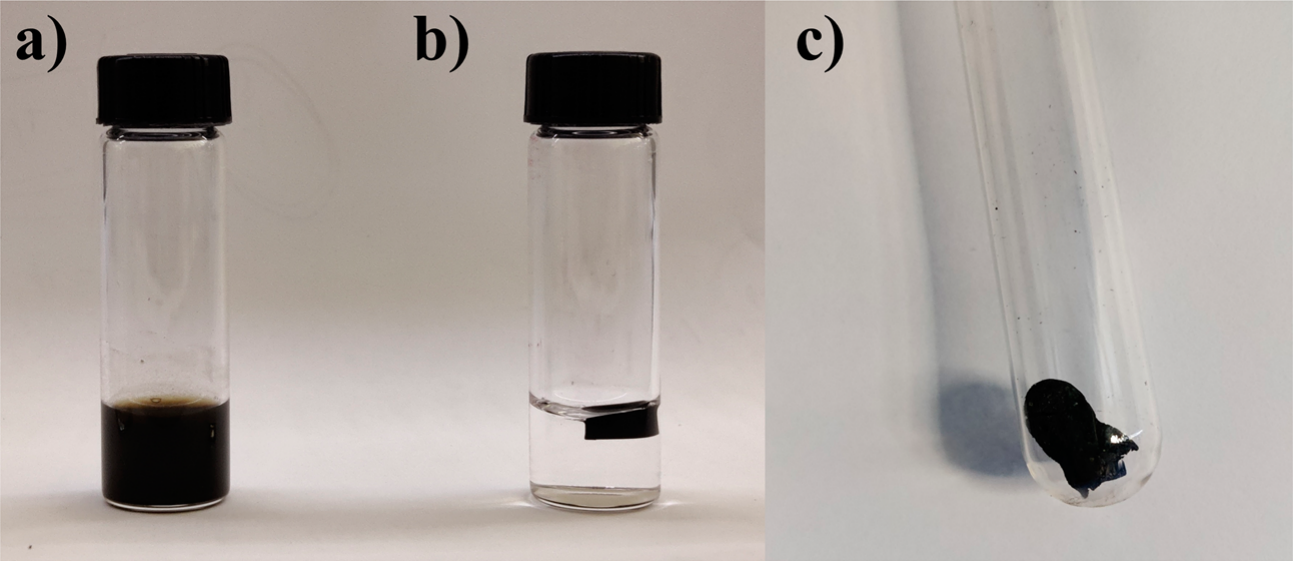
(a) Graphene oxide water dispersion; (b) rGO hydrogel after the self-assembly due to the reduction of GO layers; (c) rGOA.

### Functionalization of the Graphene Hydrogel

The rGO hydrogel was functionalized through the diazonium salt’s chemistry using the 4-nitrobenzenediazonium chloride salt (4-NBD) as a grafting agent. This compound was chosen as a grafting agent because the presence of the Ar–NO_2_ group can be easily monitored spectroscopically, which is a reliable diagnostic criterion for successful functionalization. First, 23 mg of 4-nitroaniline (4-NA) powder was solubilized with 1.5 mL of degassed H_2_O and 0.5 mL of concentrated HCl. The solution was stirred until complete solubilization. Second, 17 mg of NaNO_2_ powder was dissolved in 1.75 mL of degassed H_2_O and 0.25 mL of HCl 1M. Both solutions were then placed in an ice bath, and the resulting 2 mL of NaNO_2_ solution was slowly added drop by drop into the 4-NA solution; 4 mL of 4-NBD 42 mM solution was then obtained. The concentration of the diazonium salt used in this procedure was determined through an optimization study of the experimental conditions, carried out by Raman Microscopy and reported in paragraph S1 in the Supporting Information. Therefore, 4 mL of 4-NBD previously synthesized was injected through the reaction mixture injector (4) into the reaction chamber (5) containing a sample of the rGO hydrogel previously synthesized. The reaction mixture was kept inside the reaction chamber for 1 h at room temperature with a continuous circulating flow of 5 mL/min, activated by the peristaltic pump (6). During the functionalization step, the formation of gas bubbles from the hydrogel structure was observed; in fact, to form new C–C bonds between graphene and the diazonium salt, N_2_ is produced. After 1 h, to remove the excess of diazonium salt, the reactor was set in washing mode using the following conditions in terms of sequence and amount of solvents: 300 mL of H_2_O, 200 mL of H_2_O/EtOH (1:1), 300 mL of H_2_O (flow rate 5 mL/min). The washing flow was activated by the pneumatic pump (1), selecting the appropriate position of the switching valve system (4); the washing was collected in the waste (7). Thus, the functionalized rGO hydrogel was obtained. Finally, the sample was freeze-dried (−80 °C, overnight) to obtain the functionalized rGO aerogel (*f*-rGOA).

The implementation of a flow-based approach allows the reaction mixture to percolate through the sample in a nondestructive manner and without the need for agitation, which contributes to a homogeneous grafting process across the entire hydrogel structure. This hypothesis is supported by structural characterizations and further corroborated by a control experiment performed under static conditions that confirms the crucial role of the flow system (see Supporting Information paragraph S2).

### Characterization

GO, rGOA, and *f*-rGOA were characterized to evaluate the progressive chemical and morphological modifications (Figure 3). In order to study the surface modification on the graphene structure, micro-Raman spectroscopy was performed using a DXR3 Raman Microscope (ThermoFisher) with a laser wavelength of 780 nm as the excitation source and a ×10 objective lens. The laser power was kept at 10 mW, working in fluorescence correction mode. The collected exposure time was 1 s with 10 sample exposures. The first-order Raman spectrum was fitted with OriginLab 2018 software by the sum of 3 functions related to the D (1300–1325 cm^–1^), D″ (1500–1550 cm^–1^), and G (∼1590 cm^–1^) bands; it was found that D″ bands fit better with Gaussian functions, while D and G fit better with Voight functions. The *I*_D_/*I*_G_ ratios were calculated with the relative intensity of the D and G bands after the fitting, to eliminate the contribution due to the D″ bands on the first-order spectrum. Each sample has been tested five times at different points to have a representative value of *I*_D_/*I*_G_, fwhm of the D band, and peak position. Results are expressed as the mean.

**Figure 3:**
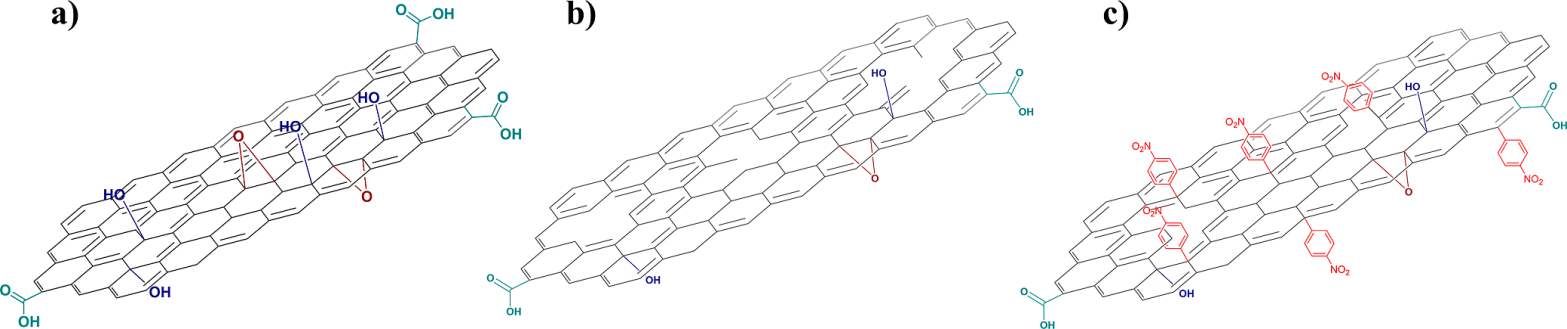
Evolution of the surface chemistry of the material after chemical treatments. (a) GO; (b) rGOA; (c) *f*-rGOA.

To evaluate the nature of the chemical functionalities, ATR–FTIR spectra were acquired using a PerkinElmer Spectrum Two FT-IR spectrometer equipped with an ATR module. All the spectra were acquired in the range of 4000–500 cm^–1^ with a resolution of 4 cm^–1^.

X-ray photoelectron spectra (XPS) were measured at a 45° takeoff angle relative to the surface sample holder, with a PHI 5000 Versa Probe II system (ULVAC-PHI, INC., base pressure of the main chamber: 1 × 10^–8^ Pa).^46,47^ Samples were excited with the monochromatized Al Kα X-ray radiation using a pass energy of 5.85 eV. The instrumental energy resolution was ≤0.5 eV. The XPS peak intensities were obtained after Shirley background subtraction. Spectral calibration was achieved by fixing the Ag 3d_5/2_ peak of a clean sample at 368.3 eV;^48^ this method turned the C 1s peak of the adventitious carbon contamination at 285.0.^46–48^ After subtraction of the background, some XP spectra were fitted with Gaussian/Lorentzian envelopes, the Lorentzian component being always lower than 20%. This process involves data refinement, based on the method of least-squares fitting, carried out until there is the highest possible correlation between the experimental spectrum and the theoretical profile. The residual or agreement factor *R*, defined by *R* = [Σ­(*F*_obs_ – *F*_calc_)^2^/Σ­(*F*_obs_)^2^]^1/2^, after minimization of the function Σ­(*F*_obs_ – *F*_calc_)^2^, converged to the value of 0.03. The atomic concentration analysis was performed by considering the relevant atomic sensitivity factors.^49^

The morphology and the microstructure of the aerogel samples are determined by scanning electron microscopy (SEM) using a ZEISS SUPRA 55VP. The specific surface area and porosity distribution were obtained from N_2_ adsorption/desorption isotherms at 77 K using a Micrometrics Tristar II 3020 (Micrometrics) apparatus. The whole aerogel samples were heat-treated (*T* = 80 °C for 24 h) before the analysis to remove adsorbed water.

### Repeatability Evaluation of the Graphene Hydrogel Synthesis

Many factors can affect the microscopic and macroscopic properties of graphene and its derivatives, such as the uneven distribution of the functional groups on the surface or the oxidation state of the carbon layers. Therefore, variable properties of the starting material can affect the repeatability of a synthetic product when a nanomaterial such as graphene is chemically processed. In our work, the graphene hydrogel was taken as a starting material, which was subsequently functionalized to obtain the final product; thus, it becomes crucial to have control over the quality of the sample that will be functionalized. For this purpose, we propose a statistical tool for processing micro-Raman data to evaluate the repeatability of the graphene hydrogel synthesis. Raman spectroscopy was chosen because it is known to be a useful tool for characterizing carbon materials and because of the amount of quantitative information that can be easily extracted from the data.^41,50,51^ For this purpose, eight independent syntheses were carried out, and for each sample, four Raman measurements were made at different points on the same sample to obtain representative values. By examining the *I*_D_/*I*_G_ ratio of all the measurements, a confidence interval (CI) was calculated to have a narrow range of acceptable values for a degree of confidence of 95%. Only samples with an *I*_D_/*I*_G_ value within the CI will proceed to the next functionalization step. The result provides a range of *I*_D_/*I*_G_ values such that hydrogel samples with values between 1.80 and 1.84 can be considered to be indistinguishable. This CI will be useful following the chemical modification; in fact, Raman analysis of graphene samples is diagnostic of the chemical changes made to the sample by comparing the *I*_D_/*I*_G_ ratios before and after. With this model, if the postfunctionalization *I*_D_/*I*_G_ differences remain within this range, no speculation will be statistically unacceptable. The full statistical workflow is available in the Supporting Information, paragraph S3.

## Result and Discussion

### Raman Analyses

Raman spectroscopy is a powerful tool for the characterization of carbon materials, especially considering that conjugated and double C=C bonds lead to high Raman intensities. Highly ordered graphite shows only two active Raman bands visible in the spectrum which are the in-phase vibration of the graphite lattice (G band) at 1575 cm^–1^, and the weak disorder band caused by the graphite edges (D band) around 1355 cm^–1^.^52^ Both the D band and the G band undergo significant changes when the surface is modified with a certain number of defects, represented by structural imperfections or the presence of tetrahedral carbon domains.^53^ A general observation is that higher disorder in graphite leads to a broader G band, as well as to a broad D band.^54^ The intensity ratios of the typical D and G bands (*I*_D_/*I*_G_) could be used as a metric of disorder in the graphene structure.^55^ Therefore, graphene-based materials show two active Raman bands in the first order spectrum, which are the G band due to the in-phase vibration of the graphitic lattice (the E_2g_ mode of the planar carbon domain) around 1590 cm^–1^, and the D band, around 1300–1325 cm^–1^, due to the symmetric A_1g_ mode.^54^

The Raman spectrum of GO (Figure 4a) shows a broad D band at 1325 cm^–1^ and a broad G band at 1596 cm^–1^; the *I*_D_/*I*_G_ ratio of 1.72 and the value of fwhm of the D band of 146 cm^–1^ are indicative of significant structural disorder due to the prominent oxidation degree.^55,56^ Following the chemical reduction of GO to obtain the rGOA, the vibration frequency of the D band decreased to 1304 cm^–1^ and the G band shifted to 1592 cm^–1^ (Figure 4b); this phenomenon could be attributed to the restoration of the π-system due to chemical reduction. Compared to GO, the *I*_D_/*I*_G_ increased to 1.83 and the fwhm of the D band decreased to 93 cm^–1^. The increased *I*_D_/*I*_G_ ratio following the chemical reduction of GO is a widely observed and discussed phenomenon in the literature, and many explanations have been given. First, this growth suggests a decrease in the average size of the planar carbon domain upon the chemical reduction of GO and can be rationalized when new graphitic domains have been created, smaller in size compared to GO but more numerous.^57,58^

**Figure 4:**
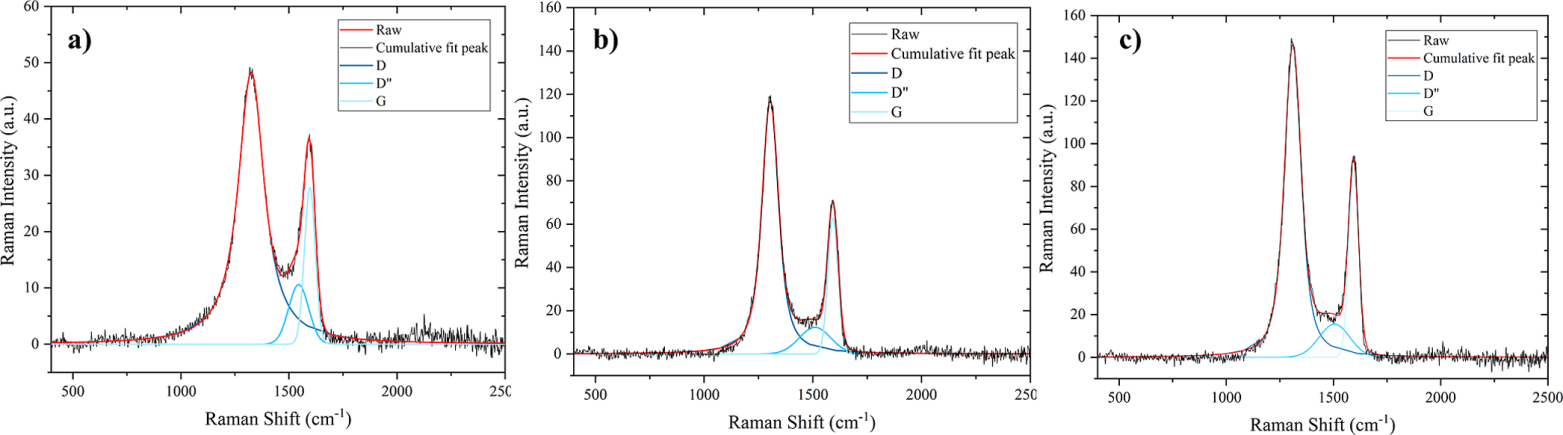
First-order Raman spectra of (a) GO; (b) rGOA; (c) *f*-rGOA.

Furthermore, it is known that structural defects are formed in the graphene sheets during the reduction of GO when the temperature surpasses 50 °C due to CO_2_ elimination.^59^ In agreement with the previous data, the decrease in fwhm of the D band from 146 cm^–1^ to 93 cm^–1^ confirms the order restoration of the graphene sheets. Interestingly, the *I*_D_/*I*_G_ ratio strongly changes following grafting of Ar-NO_2_ on the graphene structure (Figure 4c). The *I*_D_/*I*_G_ ratio decreases to 1.70, and the fwhm of the D band is 95 cm^–1^. Similar results have been reported in the literature in which the *I*_D_/*I*_G_ ratio decreases as a result of diazonium salts grafting on rGO.^60^ This suggests that, as a result of the functionalization with Ar–NO_2_, the aromatic domains of the structure begin to grow from the graphene edges.^61^ However, it cannot be excluded that this trend is due to the growth of oligomers by the diazonium salt, but this phenomenon is beyond the instrumental limits of the technique. All the results have been summarized in Table 1.

**Table 1:** First-Order Raman Spectrum Deconvolution Data Related to GO, rGOA, and *f*-rGOA Samples

parameter	GO	rGOA	*f*-rGOA
*I*_D_/*I*_G_	1.72 ± 0.02	1.83 ± 0.02	1.70 ± 0.02
*D*_position_ (cm^–1^)	1325 ± 2	1304 ± 1	1310 ± 2
*G*_position_ (cm^–1^)	1596 ± 1	1592 ± 1	1594 ± 1
*D*_fwhm_ (cm^–1^)	146 ± 1	93 ± 1	95 ± 1

Additional spatially resolved Raman measurements were carried out in different regions of the *f-*rGOH sample to assess the uniformity of the chemical functionalization. Specifically, five spectra were recorded in three distinct areas: zone 1 (upper external surface), zone 2 (lower external surface), and zone 3 (internal cross-section, sample bulk), as illustrated in Figure 5, to represent the entire sample composition. As summarized in Table 2, the consistency in the average Raman parameters obtained from the first-order spectra across all regions indicates that the flow-based functionalization method adopted leads to a homogeneous structural modification throughout the entire aerogel structure.

**Figure 5:**
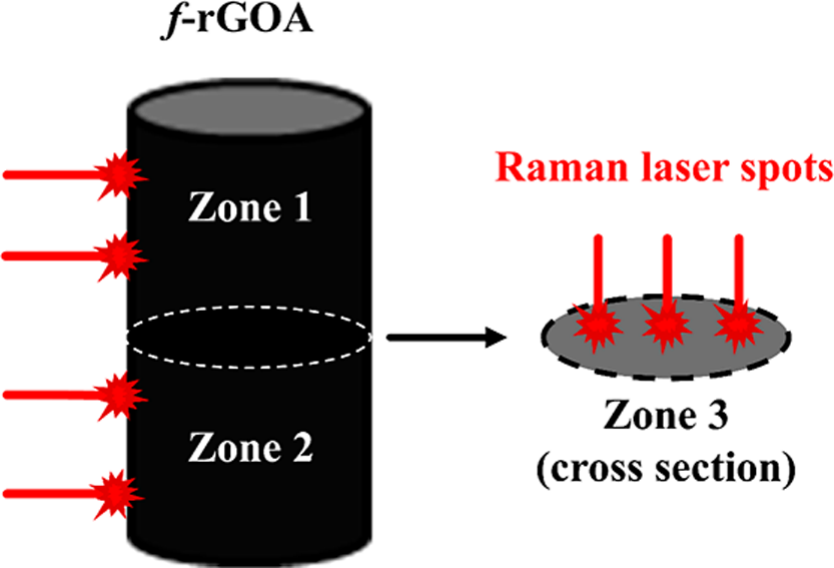
Spatially resolved Raman measurements of the *f*-rGOA sample in zone 1, zone 2, and zone 3.

**Table 2:** First-Order Raman Spectrum Deconvolution Data Related to the *f-*rGOA Sample in Different Regions

*f*-rGOA sampling area	*I*_D_/*I*_G_	*D*_position_ (cm^–1^)	*G*_position_ (cm^–1^)	*D*_fwhm_ (cm^–1^)
zone 1	1.71	1312	1595	96
	1.70	1311	1595	95
	1.69	1312	1595	96
	1.69	1312	1595	96
	1.70	1312	1595	97
**mean**	**1.70**	**1312**	**1595**	**96**
zone 2	1.69	1312	1595	95
	1.73	1308	1592	95
	1.68	1312	1594	95
	1.69	1311	1595	95
	1.67	1312	1595	94
**mean**	**1.69**	**1311**	**1594**	**95**
zone 3	1.74	1312	1595	95
	1.71	1312	1595	97
	1.68	1312	1595	95
	1.69	1312	1595	95
	1.68	1312	1596	95
**mean**	**1.70**	**1312**	**1595**	**95**
**total mean**	**1.70 ± 0.02**	**1312 ± 1**	**1595 ± 1**	**95 ± 1**

### ATR–FTIR Spectroscopy

The ATR–FTIR spectrum of graphene oxide used as a starting material, collected in ATR mode, confirms a high degree of oxidation of the structure, in agreement with the Raman data (Figure 6a). Notably, a significant peak around 3200 cm^–1^ is observed, primarily assigned to the residual water and the symmetric stretching of the O–H bonds of hydroxyl groups. The broad band around 2800 cm^–1^ corresponds to the C–H aliphatic stretching. Peaks at 1728, 1615, 1270–1220, and 1043 cm^–1^ can be attributed to C=O symmetric stretching of carbonyl and carboxyl groups, C=C symmetric stretching, C–O–C symmetric stretching of epoxy groups, and C–OH symmetric stretching, respectively.^62^

**Figure 6:**
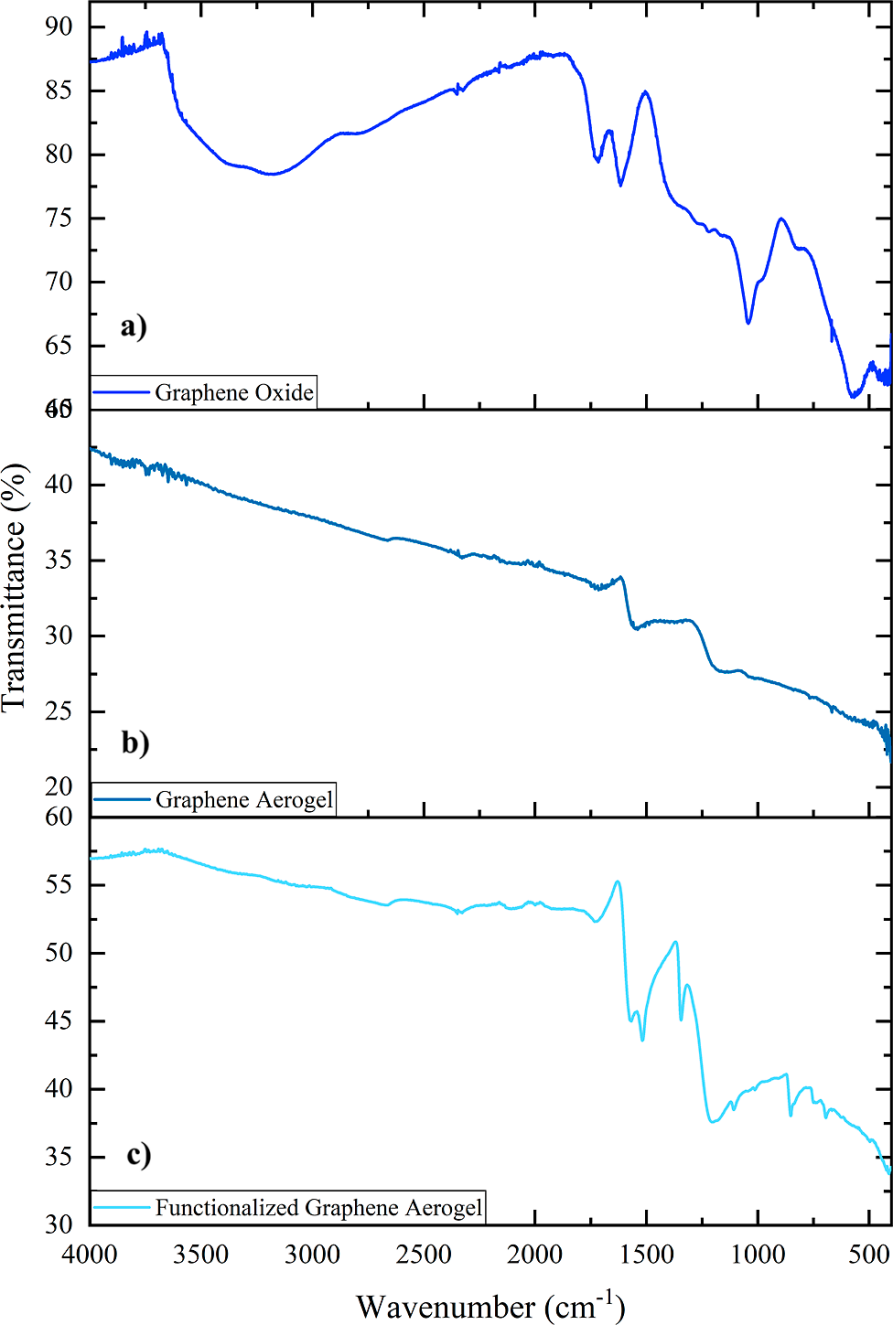
ATR–FTIR spectra of (a) GO; (b) rGOA; (c) *f*-rGOA.

The infrared spectrum of rGOA was compared with the GO spectrum to evaluate the chemical reduction step by ascorbic acid (Figure 6b). In the rGOA spectrum, only two bands around 1610 cm^–1^ and 1060 cm^–1^ are evident, corresponding to the C=C and C–OH symmetric stretching, confirming the rGO chemical structure. Importantly, no peaks attributable to the ascorbic acid are present, indicating the effectiveness of the washing step.

Finally, the *f*-rGOA spectrum was compared with the rGOA spectrum, to evaluate the functionalization step (Figure 6c). Formally, a nitrobenzene molecule was covalently added to the rGOA sample as a functional group; in fact, the *f*-rGOA spectrum shows the same bands of the rGOA with the addition of three prominent peaks at 1519 cm^–1^ and 1345 cm^–1^, attributable to the symmetric and nonsymmetric stretching of the –NO_2_ group, and a weak peak at 850 cm^–1^ related to the C–N aromatic bond.

### X-ray Photoelectron Spectroscopy

The electronic structure of graphene oxide and its derivatives was investigated by X-ray photoelectron spectroscopy. This technique gives information on the oxidation states and on the chemical environment of the studied elements and allows estimation of the surface elemental composition, once the relevant atomic sensitivity factors have been considered.^46,47,49^

Figure S4 shows the high-resolution XP spectrum of commercial graphene oxide (GO) in the C 1s binding energy region. This band is rather broad, if compared to the analogue band of the material synthesized by us, and the peak deconvolution required three Gaussian components at 284.5, 286.8, and 288.0 eV. According to the related literature,^57,63^ the signal at 284.5 eV is due to the C–C and C=C states. The band at 286.8 eV is due to the –C–OH groups, and the peak at 288.0 eV is due to the –C=O and –COO^–^ surface groups.^47,63^ The atomic concentration analysis evidenced an intensity ratio of 10:3.5:0.5. Figure S5 shows the high-resolution XP spectrum of GO in the binding energy region. The observed broad peak at 532.1 eV is largely in agreement with the already reported XPS data on GO materials.^63^ The relative XPS atomic concentration of this GO resulted in the C 71% and the O 29%, thus confirming the high oxidation of the carbon surface, in agreement with Raman and ATR–FTIR measurements. Figure 7a shows a high-resolution, sharp XP spectrum of the reduced graphene aerogel (rGOA) in the C 1s binding energy region. The peak deconvolution required five Gaussian components at 284.7, 286.0, 286.9, 288.0, and 289.1 eV. Their relative intensity ratio is about 10:1:1:0.3:0.2. According to the related literature,^47,57,63^ the signal at 284.7 eV is due to the C–C and C=C states. The band at 286.0 eV is due to the C–O–C functionality. The peak at 286.9 eV is due to the –C–OH groups. The peak at 288.0 is due to –C=O and –COO^–^ and that at 289.1 is due to –COOH.^47,63^ The atomic concentration analysis evidenced that the first band dominates the overall carbon intensity, while all the other bands are of very low intensity. Figure S6 shows the high-resolution XP spectrum of the rGOA in the O 1s binding energy region. A broad peak at about 533 eV is largely in agreement with the already reported XPS data on GO materials.^63,64^ The relative XPS atomic concentration of rGOA resulted in C 87.6% and O 12.4%. Therefore, apart from a 0.5 eV shift at higher energy, no relevant differences with respect to the GO analogue were evidenced.

**Figure 7:**
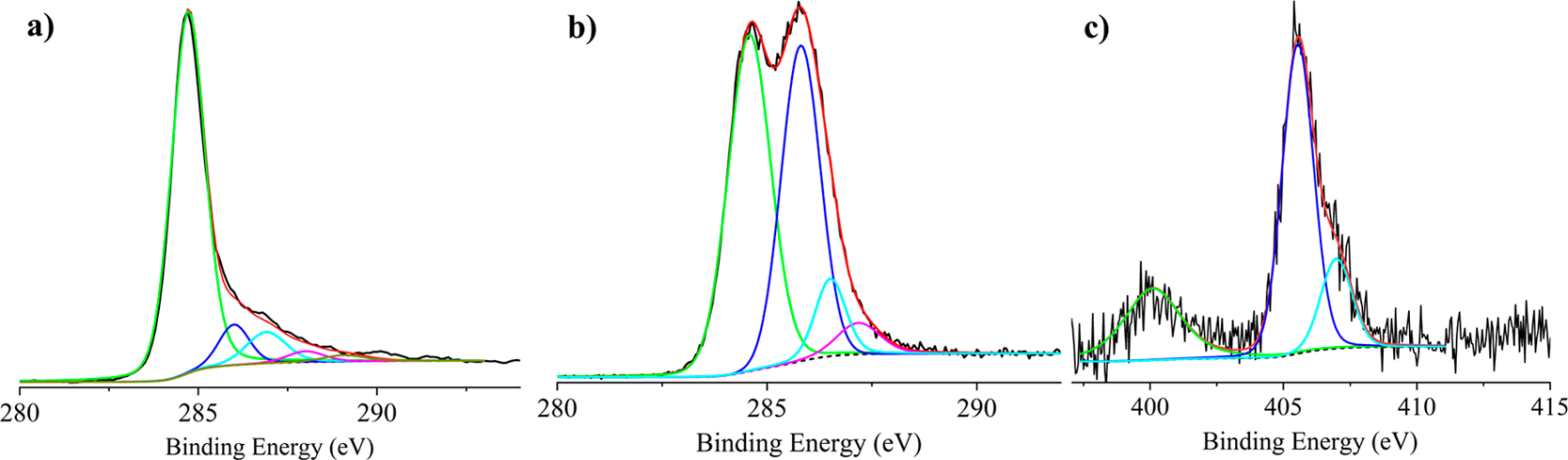
(a) Al Kα excited XPS of the rGOA in the C 1s binding energy region. The solid black line represents the experimental profile, the dotted black line represents the background, the green line represents the Gaussian component at 284.7 eV, the blue line represents the Gaussian component at 286.0 eV, the cyan line represents the Gaussian component at 286.9 eV, the magenta line represents the Gaussian component at 288.0 eV, the dark-yellow line represents the Gaussian component at 289.1 eV, and the red line, superimposed to the experimental profile, represents the sum of the Gaussian components. (b) Al Kα excited XPS of the f-rGOA in the C 1s binding energy region. The solid black line represents the experimental profile, the dotted black line represents the background, the green line represents the Gaussian component at 284.6 eV, the blue line represents the Gaussian component at 285.8 eV, the cyan line represents the Gaussian component at 286.5 eV, the magenta line represents the Gaussian component at 287.2 eV, and the red line, superimposed to the experimental profile, represents the sum of the Gaussian components. (c) Al Kα excited XPS of the *f*-rGOA in the N 1s binding energy region. The solid black line represents the experimental profile, the dotted black line represents the background, the green line represents the Gaussian component at 400.1 eV, the blue line represents the Gaussian component at 405.5 eV, the cyan lines represent the Gaussian component at 407.0 eV, and the red line, superimposed to the experimental profile, represents the sum of the Gaussian components.

This higher B.E. shift is in tune with the lower oxygen content of the rGOA with respect to that observed for the GO.

Figure 7b shows the high-resolution XP spectrum of *f*-rGOA in the C 1s binding energy region. The peak deconvolution required four Gaussian components at 284.6, 285.8, 286.5, and 287.2 eV. According to the related literature, the signal at 284.6 eV is due to the C–C and C=C states. The band at 285.8 eV is due to the C–O–C and C–N functionality,^57^ as well as to the close second carbon atoms of the –NO_2_ substituted benzene rings bonded to the graphene surface. The peak at 286.5 eV is due to some –C–OH groups. The peak at 287.2 is due to –C=O groups.^47^ Their relative intensity ratio is about 10:8:1.7:1, and the relative XPS atomic concentration of *f-*rGOA resulted in C 85.6%, O 11.4%, and N 3.0%. From these latter data, it emerges that the huge intensity increase of the band at 285.8 eV with respect to the previous cases. Therefore, the presence of the C–N functionality on the *f*-rGOA surface is now relevant, in agreement with the *f*-rGOA ATR–FTIR spectrum.

Figure 7c shows the high-resolution XP spectrum of *f*-rGOA in the N 1s binding energy region. A noisy but evident nitrogen signal is apparent. This spectrum was fitted using three Gaussian components centered at 400.1, 405.5, and 407.0 eV due to –NH_2_ groups, probably due to the unreacted 4-nitroaniline during the diazonium salt synthesis, –NO_2_ due to the grafted Ar–NO_2_, and inorganic –NO_3_ groups produced during the oxidation of the HNO_2_ in the diazotization reaction, respectively.^65^ Their relative intensity is roughly 1:4:1, thus indicating that the major nitrogen components are represented by the –NO_2_-grafted functional groups.

Figure S7 shows the high-resolution XP spectrum of the *f*-rGOA in the O 1s binding energy region. Once more, the broad peak observed at about 533 eV is largely in agreement with already reported XPS data on GO materials and precludes further speculations.^63^ Therefore, apart from a 0.5 eV shift at higher energy, no sensible differences with respect to the GO analogue were evidenced.

A comparison between the XPS C 1s signals of the three GO, rGOA, and *f*-rGOA systems suggests that the first band, due to both aliphatic and aromatic backbones, is always confined in a very narrow energy range, and in particular at (284.6 ± 0.1) eV. In all cases, this band is the most intense, and its intensity was chosen as a reference for relative intensity comparison. Considering higher binding energies, the next observed XPS band, at 285.8 eV for the *f*-rGOA and 286.0 eV for the rGOA, is absent in the GO system, thus indicating that the commercial GO system does not show any C–O–C surface functionality. In contrast, this band is huge in *f*-rGOA. Once more, this band, if present, is also pinned in a 0.2 eV narrow range. The third XPS C 1s band appears at 286.8 eV for GO (due to the –C–OH groups), 286.9 for rGOA, and 286.5 for *f*-rGOA, thus being spread in a 0.4 eV range. Its relative intensity varies a lot, being more pronounced in the commercial GO material and less pronounced in the rGOA. The fourth band is pinned at 288.0 eV for both GO and rGOA, while it appears at a lower binding energy value (287.2 eV) for the *f*-rGOA. It is clear that this band in the latter system is due to more than one carbon state and, in particular, to both −C–N and –C=O functionalities, while in both GO and rGOA, it represents ionizations of –C=O and –COO^–^ groups. Finally, the very-low-intensity XPS C 1s band at 289.1 eV, due to the –COOH group, is observed only in the case of rGOA.

### Morphological Analyses and Surface Area Measurements

The microstructure of the aerogel samples was investigated by SEM. The micrographs of the rGOA sample (Figure 8a,c) show the 3D porous micrometric network in which the multilayered rGO sheets are continuously interconnected following the self-assembly process. After functionalization, *f*-rGOA micrographs suggest that although the three-dimensional structure is retained, the pore size distribution changes (Figure 8b,d). In particular, the smaller pores decrease at the expense of larger ones. This may be due to the formation of nitrogen bubbles in the vicinity of the pores during the functionalization, which break the walls of the smaller pores to form new larger ones.

**Figure 8:**
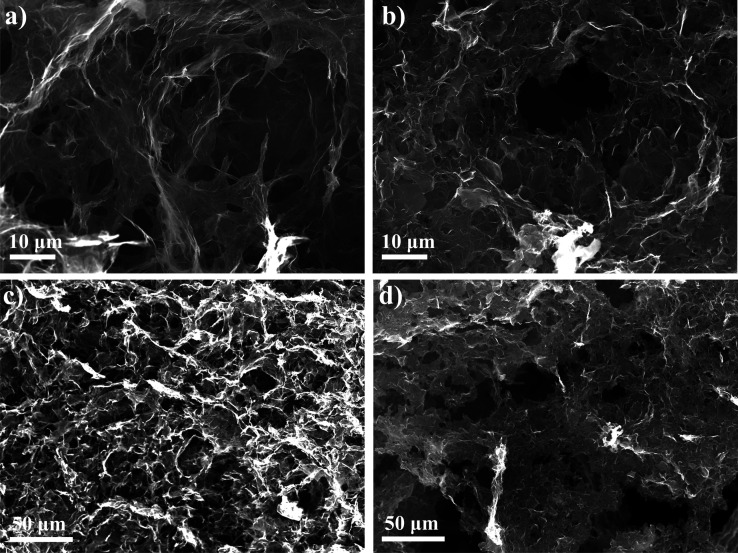
SEM images of rGOA (a,c) and *f-*rGOA (b,d).

The results obtained by Brunauer–Emmett–Teller (BET) measurements support this hypothesis (Figure 9). BET measurements were conducted on the aerogel samples to analyze the surface area and pore size distribution of these samples, and particularly, to examine any structural change due to the functionalization step. The shapes of the adsorption isotherms (Figure 9b,d) are indicative of the self-assembly of the rGO layers to form a 3D backbone. The nitrogen isotherms for both rGOA and *f*-rGOA are of the H3 type, according to the IUPAC classification, which refers to nonrigid aggregates of lamellar particles (slit-shaped pores). For the rGOA sample, the surface area is (197.0 ± 0.5) m^2^ g^–1^, while the area within the micropores is 56.5 m^2^ g^–1^. Regarding the *f*-rGOA sample, the surface area reduces to (144.9 ± 0.8) m^2^ g^–1^, with the area within the micropores declining to 20.6 m^2^ g^–1^. In addition to a decrease in surface area, it is evident from Figure 9a,c, that the functionalization process leads to changes in the distribution of pore sizes. Specifically, there is a significant reduction in micropores with a diameter of less than 2 nm, which are replaced by larger micropores with diameters ranging from 2 to 5 nm.

**Figure 9:**
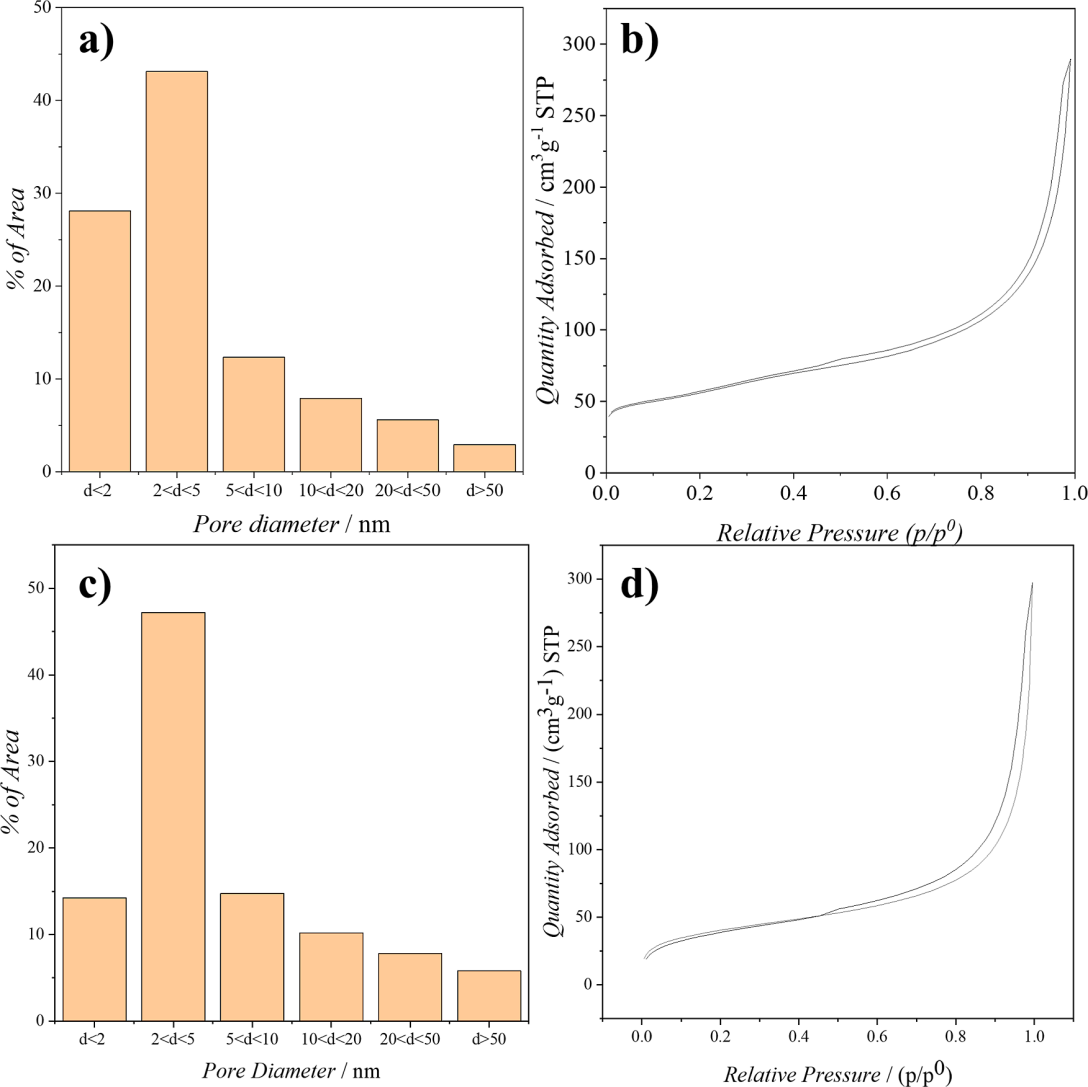
Pore size distribution and relative nitrogen isotherms of rGOA (a,b) and *f*-rGOA (c,d).

## Conclusion

In the present work, we describe a method for the covalent functionalization of three-dimensional graphene aerogels by means of diazonium salt chemistry after the synthesis of the graphene structure.

Since the 3D graphenic architecture is known to be brittle, we developed a reactor to prevent any potential damage to the structure that might occur during a conventional bulk functionalization reaction. To investigate the reaction, a well-known grafting method was selected that utilizes 4-NBD salt as a grafting agent. To evaluate the effect of the chemical functionalization, a range of spectroscopic and morphological analyses were conducted, such as XPS, ATR–FTIR, Micro-Raman, SEM, and BET. According to the spectroscopic data, it appears that the in-flow functionalization strategy has been successful in modifying the surface chemistry.

Furthermore, the proposed statistical approach could be a useful tool for assessing the repeatability of the synthesis of graphene hydrogels, and for rationalizing the Raman output for the purpose of studying the effects of chemical treatments, especially when comparing similar *I*_D_/*I*_G_ values becomes difficult.

The rapid growth of interest in three-dimensional graphene structures, such as aerogels, suggests that this strategy can be a useful tool for modifying the chemical properties of graphene aerogel structures through a simple method. In particular, the chemistry of diazonium salts proves to be very versatile for introducing heteroatoms, or more complex molecules, through the formation of covalent bonds, which are useful in various fields such as catalysis or biosensors, for example. Therefore, this approach can provide new ideas for the preparation of more complex systems in which the graphenic porous structure becomes crucial for the final application of the material. This approach can also be applied to graphene hydrogels, provided that the solvent is not removed through freeze-drying methods.

## Supplementary Material



## Notes

Diazonium salt concentration effect experiment, static conditions grafting experiment, repeatability evaluation of rGOH, and XPS supporting spectra (PDF)

## References

[1] Gorgolis G., Galiotis C. (2017). Graphene Aerogels: A Review. 2D Mater..

[2] Yang Y., Liu T., Liao Q., Ye D., Zhu X., Li J., Zhang P., Peng Y., Chen S., Li Y. (2016). A Three-Dimensional
Nitrogen-Doped Graphene Aerogel-Activated Carbon Composite Catalyst
That Enables Low-Cost Microfluidic Microbial Fuel Cells with Superior
Performance. J. Mater. Chem. A.

[3] Lin F., Cheng W. (2023). 3D Sponge Electrodes
for Soft Wearable Bioelectronics. Adv. Electron.
Mater..

[4] Xu J., Xu K., Han Y., Wang D., Li X., Hu T., Yi H., Ni Z. (2020). A 3D Porous Graphene Aerogel@GOx Based Microfluidic
Biosensor for Electrochemical Glucose Detection. Analyst.

[5] Mahmoudpour M., Dolatabadi J. E.-N., Hasanzadeh M., Soleymani J. (2021). Carbon-Based
Aerogels for Biomedical Sensing: Advances toward Designing the Ideal
Sensor. Adv. Colloid Interface Sci..

[6] Wu J., Tao K., Guo Y., Li Z., Wang X., Luo Z., Feng S., Du C., Chen D., Miao J., Norford L. K. (2017). A 3D Chemically Modified Graphene Hydrogel for Fast,
Highly Sensitive, and Selective Gas Sensor. Adv. Sci..

[7] Gao B., Feng X., Zhang Y., Zhou Z., Wei J., Qiao R., Bi F., Liu N., Zhang X. (2024). Graphene-Based
Aerogels in Water and Air Treatment: A Review. Chem. Eng. J..

[8] Cheng Z., Wang R., Wang Y., Cao Y., Shen Y., Huang Y., Chen Y. (2023). Recent Advances in
Graphene Aerogels
as Absorption-Dominated Electromagnetic Interference Shielding Materials. Carbon.

[9] Korkmaz S., Kariper I. ˙. A. (2020). Graphene
and Graphene Oxide Based Aerogels: Synthesis,
Characteristics and Supercapacitor Applications. J. Energy Storage.

[10] Dong Q., Xiao M., Chu Z., Li G., Zhang Y. (2021). Recent Progress
of Toxic Gas Sensors Based on 3D Graphene Frameworks. Sensors.

[11] Noman M. T., Amor N., Ali A., Petrik S., Coufal R., Adach K., Fijalkowski M. (2021). Aerogels for
Biomedical, Energy and
Sensing Applications. Gels.

[12] Nassar G., Daou E., Najjar R., Bassil M., Habchi R. (2021). A Review on
the Current Research on Graphene-Based Aerogels and Their Applications. Carbon Trends.

[13] Kumar S. S., Aparna A., Sreehari H., Aathira U., Lekshmi A. G., Aiswarya A. S., Sooryalekshmi M., Navami J. S., Saritha A. (2024). A Comprehensive
Review on Synthesis and Applications of Graphene Aerogel-Based Nanocomposites. J. Sol-Gel Sci. Technol..

[14] Enterría M., Figueiredo J. L. (2016). Nanostructured Mesoporous Carbons:
Tuning Texture and
Surface Chemistry. Carbon.

[15] Allahbakhsh A., Gadegaard N., Ruiz C. M., Shavandi A. (2024). Graphene-Based
Engineered
Living Materials. Small Methods.

[16] Leng X., Vazquez R. J., McCuskey S. R., Quek G., Su Y., Nikolaev K. G., Costa M. C. F., Chen S., Chen M., Yang K., Zhao J., Lin M., Chen Z., Bazan G. C., Novoselov K. S., Andreeva D. V. (2023). Bacteria-Loaded
Graphene Bioanode for Renewable Energy Generation. Carbon.

[17] Plata-Gryl M., Castro-Muñoz R., Boczkaj G. (2023). Chemically Reduced Graphene Oxide
Based Aerogels - Insight on the Surface and Textural Functionalities
Dependent on Handling the Synthesis Factors. Colloids Surf., A.

[18] Dreyer D. R., Park S., Bielawski C. W., Ruoff R. S. (2010). The Chemistry of
Graphene Oxide. Chem. Soc. Rev..

[19] Guo S., Garaj S., Bianco A., Ménard-Moyon C. (2022). Controlling
Covalent Chemistry on Graphene Oxide. Nat. Rev.
Phys..

[20] Joshi D. J., Koduru J. R., Malek N. I., Hussain C. M., Kailasa S. K. (2021). Surface
Modifications and Analytical Applications of Graphene Oxide: A Review. TrAC, Trends Anal. Chem..

[21] Chua C. K., Pumera M. (2013). Covalent Chemistry
on Graphene. Chem. Soc. Rev..

[22] Bai, J. ; Mei, J. Architectural and Chemical Aspects of 3D Graphene for Emerging Applications. In 3D Graphene: Fundamentals, Synthesis, and Emerging Applications; Springer, 2023; pp 59–74.

[23] Tang C., Wang H.-F., Huang J.-Q., Qian W., Wei F., Qiao S.-Z., Zhang Q. (2019). 3D Hierarchical Porous Graphene-Based
Energy Materials: Synthesis, Functionalization, and Application in
Energy Storage and Conversion. Electrochem.
Energy Rev..

[24] Feng Q., Li T., Sui Y., Xiao B., Wang T., Sun Z., Qi J., Wei F., Meng Q., Ren Y., Xue X. (2021). Facile Synthesis
and First-Principles Study of Nitrogen and Sulfur Dual-Doped Porous
Graphene Aerogels/Natural Graphite as Anode Materials for Li-Ion Batteries. J. Alloys Compd..

[25] Bin Y., Liang Q., Luo H., Chen Y., Wang T. (2023). One-Step Synthesis
of Nitrogen-Functionalized Graphene Aerogel for Efficient Removal
of Hexavalent Chromium in Water. Environ. Sci.
Pollut. Res..

[26] Shabangoli Y., Rahmanifar M. S., Noori A., El-Kady M. F., Kaner R. B., Mousavi M. F. (2019). Nile Blue Functionalized Graphene Aerogel as a Pseudocapacitive
Negative Electrode Material across the Full PH Range. ACS Nano.

[27] Xu Q., Jia H., Duan X., Lu L., Tian Q., Chen S., Xu J., Jiang F. (2020). Label-Free Electrochemical Immunosensor for the Detection
of Prostate Specific Antigen Based Three-Dimensional Au Nanoparticles/MoS2-Graphene
Aerogels Composite. Inorg. Chem. Commun..

[28] Fu K., Zhao J., Liu F., Wu L., Jin Z., Yang Y., Qiao J., Wang Z., Wang F., Liu J. (2023). Enhanced Electromagnetic Wave Absorption of Nitrogen-Doped Reduced
Graphene Oxide Aerogels with LaFeO3 Cluster Modifications. Carbon.

[29] Liu Z., Zhou H., Huang Z., Wang W., Zeng F., Kuang Y. (2013). Graphene Covalently
Functionalized with Poly­(p-Phenylenediamine)
as High Performance Electrode Material for Supercapacitors. J. Mater. Chem. A.

[30] Ullah S., Hasan M., Ta H. Q., Zhao L., Shi Q., Fu L., Choi J., Yang R., Liu Z., Rümmeli M. H. (2019). Synthesis
of Doped Porous 3D Graphene Structures by Chemical Vapor Deposition
and Its Applications. Adv. Funct. Mater..

[31] Sun Y., Wu Q., Zhang K., Liu Y., Liang X., Xiang H. (2022). A High Areal
Capacity Sodium-Ion Battery Anode Enabled by a Free-Standing Red Phosphorus@N-Doped
Graphene/CNTs Aerogel. Chem. Commun..

[32] Peng K., Wang Y., Liu F., Wan P., Wang H., Niu M., Su L., Zhuang L., Qin Y. (2023). Hierarchical SiC–Graphene
Composite Aerogel-Supported Ni–Mo–S Nanosheets for Efficient
PH-Universal Electrocatalytic Hydrogen Evolution. ACS Appl. Mater. Interfaces.

[33] Sun Z., Zhao K., Yang H., Liang J., Chen Z., Feng J., Jiang Y., Li L., Hu Y., Feng J. (2024). Research Progress on Modification of Aerogels by Chemical Vapor Deposition. Langmuir.

[34] González, M. C. R. ; Mali, K. S. ; De Feyter, S. Covalent Modification of Graphite and Graphene Using Diazonium Chemistry. In Aryl Diazonium Salts and Related Compounds: Surface Chemistry and Applications; Springer, 2022; pp 157–181.

[35] Paulus G. L. C., Wang Q. H., Strano M. S. (2013). Covalent
Electron Transfer Chemistry
of Graphene with Diazonium Salts. Acc. Chem.
Res..

[36] Gautier C., López I., Breton T. (2021). A Post-Functionalization Toolbox
for Diazonium (Electro)-Grafted Surfaces: Review of the Coupling Methods. Mater. Adv..

[37] Aryl Diazonium Salts and Related Compounds. Physical Chemistry in Action; Chehimi, M. M. , Pinson, J. , Mousli, F. , Eds.; Springer International Publishing: Cham, 2022.

[38] Downard A.
J. (2000). Electrochemically
Assisted Covalent Modification of Carbon Electrodes. Electroanalysis.

[39] Rebuttini V., Fazio E., Santangelo S., Neri F., Caputo G., Martin C., Brousse T., Favier F., Pinna N. (2015). Chemical Modification
of Graphene Oxide through Diazonium Chemistry and Its Influence on
the Structure–Property Relationships of Graphene Oxide–Iron
Oxide Nanocomposites. Chem.—A Eur. J..

[40] Wang L., Ye Y., Lu X., Wu Y., Sun L., Tan H., Xu F., Song Y. (2013). Prussian Blue
Nanocubes on Nitrobenzene-Functionalized
Reduced Graphene Oxide and Its Application for H2O2 Biosensing. Electrochim. Acta.

[41] Wróblewska A., Dużyńska A., Judek J., Stobiński L., Żerańska K., Gertych A. P., Zdrojek M. (2017). Statistical
Analysis of the Reduction Process of Graphene Oxide Probed by Raman
Spectroscopy Mapping. J. Phys.: Condens. Matter.

[42] Englert J. M., Vecera P., Knirsch K. C., Schäfer R. A., Hauke F., Hirsch A. (2013). Scanning-Raman-Microscopy for the
Statistical Analysis of Covalently Functionalized Graphene. ACS Nano.

[43] Fernández-Merino M. J., Guardia L., Paredes J. I., Villar-Rodil S., Solís-Fernández P., Martínez-Alonso A., Tascón J. M. D. (2010). Vitamin C Is an Ideal Substitute for Hydrazine in the
Reduction of Graphene Oxide Suspensions. J.
Phys. Chem. C.

[44] Chen W., Yan L. (2011). In Situ Self-Assembly of Mild Chemical Reduction Graphene for Three-Dimensional
Architectures. Nanoscale.

[45] Longo A., Palomba M., Carotenuto G. (2020). Green Solid-State Chemical Reduction
of Graphene Oxide Supported on a Paper Substrate. Coatings.

[46] Matthew, J. Surface Analysis by Auger and X-Ray Photoelectron Spectroscopy; Briggs, D. and Grant, J. T., Eds.; IMPublications, Chichester, UK and SurfaceSpectra; Wiley Online Library, 2004; p 1647.

[47] Gulino A. (2013). Structural
and Electronic Characterization of Self-Assembled Molecular Nanoarchitectures
by X-Ray Photoelectron Spectroscopy. Anal. Bioanal.
Chem..

[48] Greczynski G., Hultman L. (2020). Compromising Science by Ignorant Instrument Calibration—Need
to Revisit Half a Century of Published XPS Data. Angew. Chem..

[49] Li S., Zhang H., Liu Z., Xu J., Fan G., Li W., Li Q., Hu X., Jing G. (2022). Auger Electron Spectroscopy
(AES) and X-Ray Photoelectron Spectroscopy (XPS) Profiling of Self
Assembled Monolayer (SAM) Patterns Based on Vapor Deposition Technique. Appl. Sci..

[50] Goldie S. J., Bush S., Cumming J. A., Coleman K. S. (2020). A Statistical Approach
to Raman Analysis of Graphene-Related Materials: Implications for
Quality Control. ACS Appl. Nano Mater..

[51] Muzyka R., Drewniak S., Pustelny T., Sajdak M., Drewniak Ł. (2021). Characterization
of Graphite Oxide and Reduced Graphene Oxide Obtained from Different
Graphite Precursors and Oxidized by Different Methods Using Raman
Spectroscopy Statistical Analysis. Mater..

[52] Tuinstra F., Koenig J. L. (1970). Raman Spectrum of Graphite. J.
Chem. Phys..

[53] Ferrari A.
C., Robertson J. (2000). Interpretation
of Raman Spectra of Disordered and Amorphous
Carbon. Phys. Rev. B:Condens. Matter Mater.
Phys..

[54] Kudin K. N., Ozbas B., Schniepp H. C., Prud’homme R. K., Aksay I. A., Car R. (2008). Raman Spectra of Graphite Oxide and
Functionalized Graphene Sheets. Nano Lett..

[55] Mohan V. B., Nieuwoudt M., Jayaraman K., Bhattacharyya D. (2017). Quantification
and Analysis of Raman Spectra of Graphene Materials. Graphene Technol..

[56] Ferrari A. C. (2007). Raman Spectroscopy
of Graphene and Graphite: Disorder, Electron–Phonon Coupling,
Doping and Nonadiabatic Effects. Solid State
Commun..

[57] Stankovich S., Dikin D. A., Piner R. D., Kohlhaas K. A., Kleinhammes A., Jia Y., Wu Y., Nguyen S. T., Ruoff R. S. (2007). Synthesis of Graphene-Based
Nanosheets via Chemical Reduction of Exfoliated Graphite Oxide. Carbon.

[58] Liu H., Zhang L., Guo Y., Cheng C., Yang L., Jiang L., Yu G., Hu W., Liu Y., Zhu D. (2013). Reduction of Graphene Oxide to Highly Conductive Graphene by Lawesson’s
Reagent and Its Electrical Applications. J.
Mater. Chem. C.

[59] Eigler S., Dotzer C., Hirsch A., Enzelberger M., Müller P. (2012). Formation and Decomposition of CO
2 Intercalated Graphene
Oxide. Chem. Mater..

[60] Chiticaru E. A., Pilan L., Ioniţă M. (2022). Electrochemical Detection
Platform Based on RGO Functionalized with Diazonium Salt for DNA Hybridization. Biosensors.

[61] Jiang D., Sumpter B. G., Dai S. (2006). How Do Aryl
Groups Attach to a Graphene
Sheet?. J. Phys. Chem. B.

[62] Jaworski S., Wierzbicki M., Sawosz E., Jung A., Gielerak G., Biernat J., Jaremek H., Łojkowski W., Woźniak B., Wojnarowicz J., Mazurkiewicz-Pawlicka L. A. M., Łojkowski M., Kurantowicz N., Chwalibog A., Kurantowicz N., Chwalibog A. (2018). Graphene Oxide-Based
Nanocomposites Decorated with Silver Nanoparticles as an Antibacterial
Agent. Nanoscale Res. Lett..

[63] Giofrè S., Tiecco M., Celesti C., Patanè S., Triolo C., Gulino A., Spitaleri L., Scalese S., Scuderi M., Iannazzo D. (2020). Eco-Friendly 1,3-Dipolar
Cycloaddition Reactions on Graphene Quantum Dots in Natural Deep Eutectic
Solvent. Nanomaterials.

[64] Zimbone M., Cacciato G., Boutinguiza M., Gulino A., Cantarella M., Privitera V., Grimaldi M. G. (2019). Hydrogenated Black-TiOx: A Facile
and Scalable Synthesis for Environmental Water Purification. Catal. Today.

[65] Graf N., Yegen E., Gross T., Lippitz A., Weigel W., Krakert S., Terfort A., Unger W. E. S. (2009). XPS and NEXAFS
Studies of Aliphatic and Aromatic Amine Species on Functionalized
Surfaces. Surf. Sci..

