# Supplementary material for: A Novel Flow Chemistry Approach to Covalent Functionalization of 3D Graphene Aerogels
Source: ACS Omega. 2025 Jul 7;10(28):30576–86. doi: 10.1021/acsomega.5c02481 (PMC12290618; doi:10.1021/acsomega.5c02481)
Supplement: Supplementary file 1 [file ao5c02481_si_001.pdf]

SUPPORTING INFORMATIONS: A Novel Flow Chemistry Approach to Covalent Functionalization of 3D Graphene Aerogels

Antonino Biagio Carbonaro[a], Valentina Greco[a], Valentina Pifferi[b], Luigi Falciola[b,c], Enrico Ciliberto[a], Antonino Gulino[a], Alessandro Giuffrida[a].

[a] *Department of Chemical Sciences, Università degli Studi di Catania, Viale A. Doria 6, 95100, Catania, Italy*

[b] *Department of Chemistry, Università degli Studi di Milano, via Golgi 19, 20133, Milano, Italy.*

[c] *Consorzio Interuniversitario Nazionale per la Scienza e Tecnologia dei Materiali (INSTM), Via G. Giusti, 9 50121 Firenze, Italy*

S1. Diazonium salt concentration effect

The effect of concentration on the functionalization reaction of rGOA was evaluated by Raman Microscopy. To this end, three different reactions were carried out under the same conditions described in the main text (see "Functionalization of graphene hydrogel"), except for the concentrations of the 4-nitroaniline and sodium nitrite. This variation was intended to control the final concentration of the diazonium salt formed in the reaction mixture injected into the reactor. The concentrations were not chosen arbitrarily but rather estimated based on an approximate ratio between the number of carbon atoms present in the graphene hydrogel sample, and the number of diazonium salt molecules formed during diazotization. The reaction between 4-nitroaniline and sodium nitrite was assumed to proceed quantitatively. The number of carbon atoms in the sample was estimated based on the initial weight of graphene oxide (GO) used in the synthesis. Specifically, 750 µL of GO dispersion contains approximately 2.25 mg of GO, of which—based on relative XPS elemental analysis—only 71% is carbon. Assuming negligible mass loss due to CO_2 evolution during the reduction of GO to form the hydrogel, the amount of carbon available for the grafting reaction in the rGOA sample is 1.6 mg, corresponding to approximately 8.02×10^{19} carbon atoms. This estimated value was used to select the concentrations employed to test the effect of the diazonium salt concentration, specifically by evaluating the ratio between the number of carbon atoms in the graphene hydrogel sample and the number of available diazonium salt molecules. Accordingly, "Graphene/Diazonium salt" ratios of 4:1, 1:1, and 1:6 were selected as investigative experimental conditions, and the corresponding concentrations of 4-nitroaniline and sodium nitrite were adjusted to yield final diazonium salt concentrations of 10 mM, 42 mM, and 250 mM, respectively. Raman analyses were then performed on the corresponding *f*-rGOA samples, and the key diagnostic indicators of the reaction—namely the I_D/I_G ratio and the FWHM of the D band—were compared.

Figure S1 presents the Raman output of the *f*-rGOA samples after data processing, obtained at different concentrations of the 4-NBD salt. Notably, the 1:1 molar ratio—corresponding to a 42 mM concentration—was selected as the optimal functionalization condition. This choice was based on the

most significant structural modifications observed in the final *f*-rGOA product, as evidenced by variations in both the I_D/I_G ratio and the FWHM of the D band when compared to the unfunctionalized rGOH structure (for the full interpretation see "Raman Analyses" in the main text).

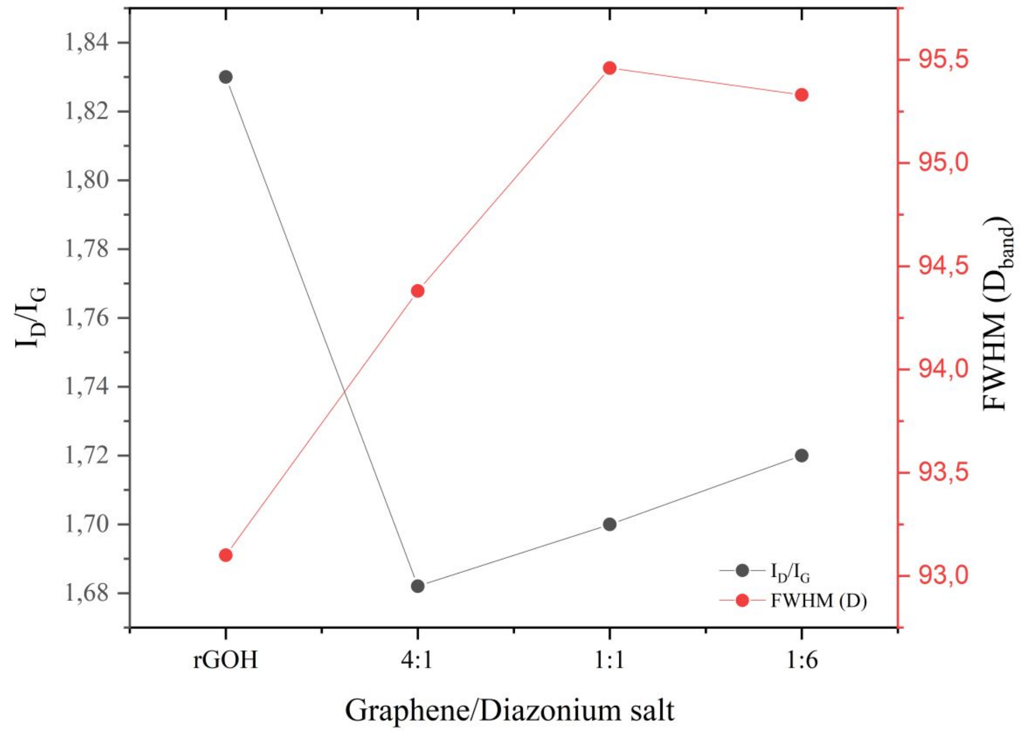

Figure S1: Trend of the I_D/I_G ratio and the FWHM of the D band for the *f*-rGOH samples functionalized at different concentrations of 4-NBD salt.

S2: Static condition grafting of rGOH

The same grafting reaction was carried out under the conditions described in the Experimental Section, with the only difference being the use of a static setup instead of the flow reactor. Specifically, the hydrogel sample was placed inside a test tube containing 4 mL of the freshly prepared diazonium salt solution. As shown in Figure S 2a, the sample was immediately immersed in the reaction mixture, and the appearance of gas bubbles (nitrogen) emerging from the structure confirmed that the grafting reaction was taking place. After 5 minutes, a fluorescent yellow gelatinous mass began to form around the hydrogel (Figure S 2b), which evolved into an orange flocculent precipitate covering the entire hydrogel sample after 10 minutes (Figure S 2c). Although this material was not characterized, it is likely the result of a rapid dendrimer polymerization of the diazonium salt, well known phenomenon in literature during the grafting mechanism[8,9]. A plausible explanation for the observed phenomenon may lie in the limited diffusion of the reaction mixture within the hydrogel structure. In the absence of convective flow, the reagents remain predominantly confined to the surface, leading to the formation of steep concentration gradients and consequently favouring the rapid polymerization of the diazonium salt, causing structural damages on the external surface of the

sample. This hypothesis is supported by the observation that, when the reaction mixture is flowed through the system using a flow reactor, the solution remains completely clear and free of precipitates even after one hour of reaction. Furthermore, no structural damage was observed as a result of the flow reaction.

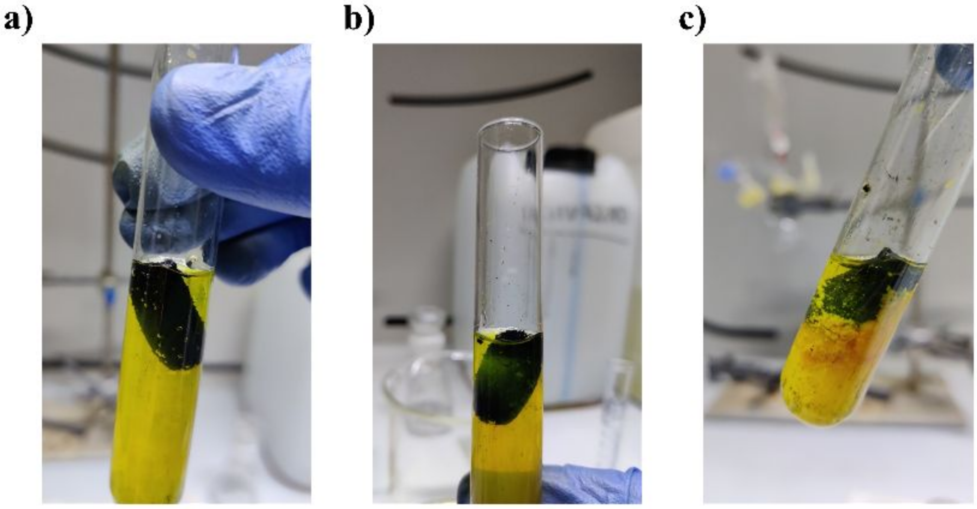

Figure S2: Grafting reaction of rGOH performed in static condition; **a)** rGOH immersed in the 4-NBD salt solution. **b)** rGOH after 5 minutes; the fluorescent yellow gelatinous mass starts to appear on the hydrogel surface. **c)** yellow flocculate likely resulting from the polymerization of the diazonium salt around the graphene hydrogel.

S3. Repeatability evaluation of the graphene hydrogel synthesis

The repeatability of the graphene hydrogel synthesis was evaluated by studying the I_D/I_G ratio to assess the effect of the chemical reduction by the ascorbic acid. In this regard, eight graphene hydrogel samples (labelled from 1 to 8) were studied by making four measurements for each sample. The mean ($\bar{x}_j$), the sample standard deviation (s) and the variance (s^2) of the I_D/I_G value were calculated for each group of samples; results are shown in **Table S1**.

Sample	1	2	3	4	5	6	7	8
	1.83	1.87	1.81	1.80	1.81	1.83	1.81	1.75
I_D/I_G	1.83	1.87	1.87	1.84	1.85	1.83	1.85	1.79
value	1.78	1.87	1.82	1.86	1.85	1.83	1.77	1.80
	1.78	1.83	1.84	1.84	1.83	1.86	1.79	1.80
$\bar{x}_j$	1.81	1.86	1.84	1.84	1.84	1.84	1.81	1.79
s	0.029	0.020	0.026	0.025	0.019	0.015	0.034	0.024
s^2	0.00083	0.0004	0.0007	0.00063	0.00037	0.00023	0.00117	0.00057

Table S1: Experimental I_D/I_G values for the eight graphene hydrogel samples.

S 3.1. Upper limit variance outlier test: Cochran's C test

The Cochran's C test, preferred for normally distributed data and equal size groups[1], also suggested by IUPAC[23], is carried out to check the presence of outliers in the upper limit of the variances of the different groups ($g = 8$). In our case, to check whether the population of variances is normally distributed, the Shapiro-Wilk test was performed according to the following formula[45]:

$$W = \frac{\left(\sum_{i=1}^{n} a_i x_{(i)}\right)^2}{\sum_{i=1}^{n} (x_{(i)} - \bar{x})^2} \quad \textbf{(Eq. S1)}$$

where $n = 8$ is the number of observations, x_i is the value of each variance and, a_i are the tabulated coefficients for the test[4]. For this dataset, $W = 0.948$, greater than the tabulated value $W_{n=8;\,\alpha=0.05}$ $= 0.818$; therefore, at the $p = 1 - \alpha = 0.95$ level, the variances were significantly drawn from a normally distributed population[4].

The *C value* given by the ratio between the maximum variance (s_{max}^2), and the sum of the variances of each group was calculated and compared with the tabulated critical value $C_{p,v,g}$, ($p = 1 - \alpha = 0.95; v = n - 1$, where n is the number of measurements for each group; $g = 8$) with the following criterion: $C \leq C_{p,v,g}$

$$C = \frac{s_{max}^2}{\sum_{j=1}^{g} s_j^2} = \frac{1.2 \times 10^{-3}}{4.5\ x \times 10^{-3}} = 0.23$$

$$C_{p=0,v=3,g=8} = 0.44$$

Here, $C \leq C_{p,v,g}$ is fulfilled, and the s_{max}^2 is not an outlier. Then, the *minimum variance outlier test* was performed to check the minimum variance (s_{min}^2) according to the following criterion: $C \geq C_{p,v,g}$

$$C = \frac{s_{min}^2}{\sum_{j=1}^{g} s_j^2} = \frac{2.30 \times 10^{-4}}{4.9\ \times 10^{-3}} = 4.60 \times 10^{-2}$$

$$C_{p=0,v=3,g=8} = 3.87 \times 10^{-3}$$

Again, $C \geq C_{p,v,g}$ was verified, and s_{min}^2 is not an outlier.

S 3.1.2. Checking for outliers in the data set for the averages: Huber test

Before processing the data, it is necessary to check whether there are outliers in the data set. Unlike the Grubbs and Dixon tests, the Huber test, which is based on the median, is not affected by the distance of the extreme values of an ordered set of data from its median[6]. In this case, the dataset is represented by the $\bar{x}_j$ values reported in Table 1.

Firstly, we calculated the median value $\tilde{x}$ of the $\bar{x}_j$ values ($\tilde{x} = 1.84$) and then, the differences $|r_j|$ $= |\bar{x}_j - \tilde{x}|$ are calculated as follows:

$$|r_1| = |\bar{x}_1 - \tilde{x}|, |r_2| = |\bar{x}_2 - \tilde{x}|, |r_3| = |\bar{x}_3 - \tilde{x}|,..., |r_n| = |\bar{x}_n - \tilde{x}| \quad \textbf{(Eq. S2)}$$

Secondly, the Median Absolute Deviation (MAD) $[\tilde{r}]$ of $|r_j|$ are obtained ($[\tilde{r}] = 0.01$), and the results are shown in Table S 5.1.2. Finally, according to the Huber test, the data is accepted if the following relationship is fulfilled:

$$|\bar{x}_j - \tilde{x}| = |r_j| \leq 4.5 \cdot [\tilde{r}] \quad \textbf{(Eq. S3)}$$

After the verification in each group, no outliers were found for $\bar{x}_j$ values; positive results were marked with "✓" in **Table S 2**.

**Group**	**1**	**2**	**3**	**4**	**5**	**6**	**7**	**8**
$\bar{x}_j$	1.81	1.86	1.84	1.84	1.84	1.84	1.81	1.79
$\lvert r_j\rvert = \lvert\bar{x}_j - \tilde{x}\rvert$	0.03	0.03	0	0	0	0	0.03	0.03
$\lvert r_j\rvert \leq 4.5 \cdot [\tilde{r}]$	✓	✓	✓	✓	✓	✓	✓	✓

Table S2: Huber test values

S 3.1.3. Analysis of variance (ANOVA) and confidence interval calculation for the total mean

The analysis of variance (ANOVA) at one level was performed to test the variability between the different groups of measurements[7]. The null hypothesis of the test is that if the within-group variance is not significantly different from the between-group variance, then there is no statistically significant variation in I_D/I_G mean values between each group, due to the hydrogel preparation.

For this purpose, the sum of squares *between* groups (SS_B) and *within* groups (SS_W) were calculated according to the following formulas:

$$SS_B = \sum_{j=1}^{g} n_j(\bar{x}_j - \bar{x}_{tot})^2 \quad \textbf{(Eq. S4)}$$

$$SS_W = \sum_{j=1}^{g}\sum_{i=1}^{n_j} (x_{ij} - \bar{x}_j)^2 \quad \textbf{(Eq. S5)}$$

where n_j is the number of replicates in each group and $\bar{x}_{tot} = 1.82$ is the total mean.

For our dataset, $SS_B = 0.016$ ($v = g - 1 = 7$) and $SS_W = 0.015$ ($v = n - g = 24$). For deciding on the acceptance of the null hypothesis, an *F*-test is performed according to the **(Eq. S6):**

$$F_{calc} = \frac{\frac{SS_B}{g-1}}{\frac{SS_W}{n-g}} \quad \textbf{(Eq. S6)}$$

Since the calculated *F-value* ($F_{calc} = 3.81$) is higher than the tabulated one $F_{tab} = 2.42$ ($p = 1 - \alpha = 0{,}95, f_1 = 7; f_2 = 24$), we cannot accept the null hypothesis; therefore, the differences in the mean I_D/I_G values obtained for each individual group are not due to random errors, but are affected by the hydrogel preparation. Thus, the total variance ($s_{\bar{x}}^2$) was calculated from the total sum of squares (SS_t), according to (Eq. S7), divided by the degrees of freedom of this variance ($v = n - 1 = 32 - 1 = 31$):

$$SS_t = SS_B + SS_W = 0.031 \quad \textbf{(Eq. S7)}$$

$$s_{\bar{x}}^2 = \frac{SS_t}{n-1} = \frac{0.031}{31} = 1 \times 10^{-3}$$

Finally, the confidence interval related to the total mean coming from the $g = 8$ groups is expressed as follows:

$$x = \bar{x}_{tot} \pm \frac{s_{\bar{x}}}{\sqrt{g}} \cdot t_{p=1-\alpha=0.95;v=31} \quad \textbf{(Eq. S8)}$$

$$x = (1.82 \pm 0.02)$$

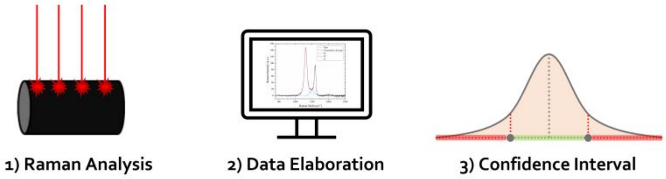

Figure S3: Statistical workflow representation

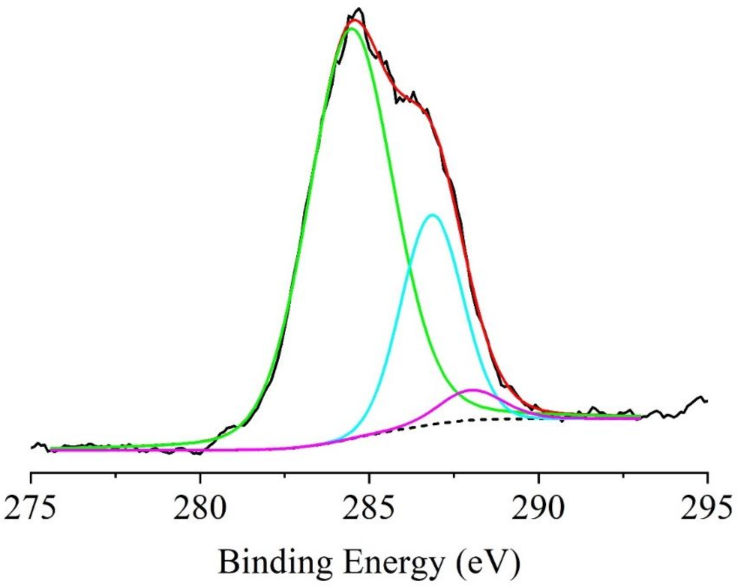

Figure S4. Al Kα excited XPS of the GO in the C 1s binding energy region. The solid black line represents the experimental profile, the dotted black line represents the background, the green line, represents the Gaussian component at 284.5 eV, the cyan line represents the Gaussian component at 286.8 eV, the magenta line represent Gaussian component at 288.0 eV and the red line, superimposed to the experimenyal profile, represents the sum of the Gaussian components.

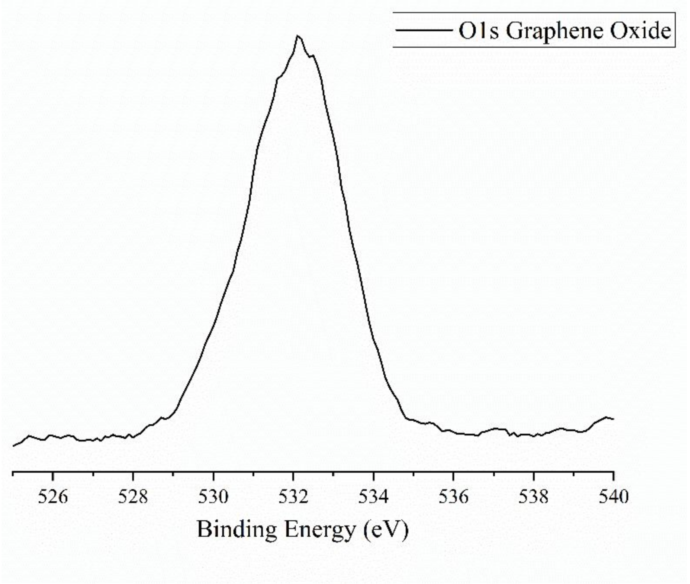

Figure S5. Al Kα excited XPS of the GO in the O 1s binding energy region.

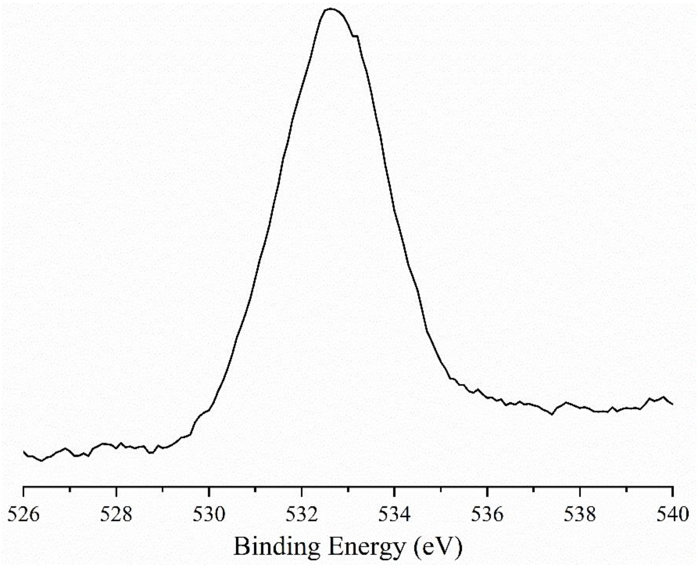

Figure S6. Al Kα excited XPS of the rGOA in the O 1s binding energy region.

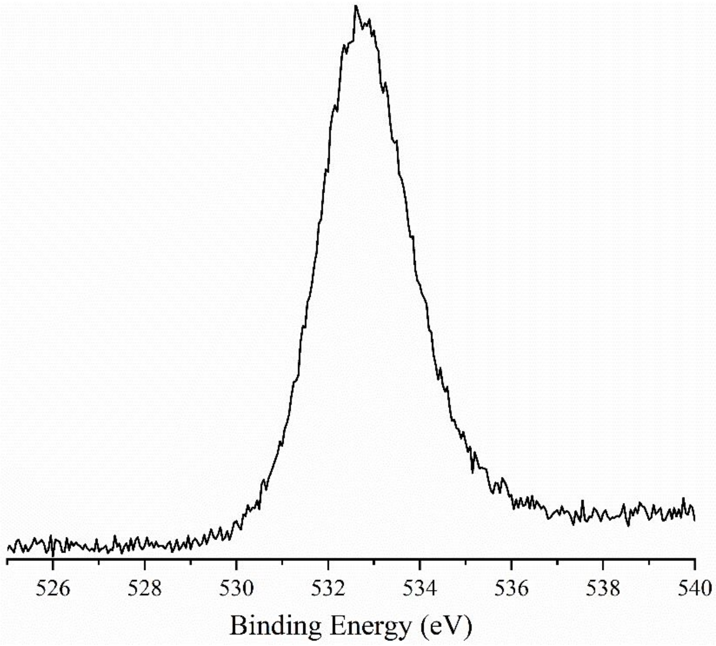

Figure S7. Al Kα excited XPS of the *f*-rGOA in the O 1s binding energy region.

REFERENCES

(1) Zhou, Y.; Zhu, Y.; Wong, W. K. Statistical Tests for Homogeneity of Variance for Clinical Trials and Recommendations. *Contemp. Clin. Trials Commun.* **2023**, *33*, 101119.

(2) Horwitz, W. Protocol for the Design, Conduct and Interpretation of Method-Performance Studies: Revised 1994 (Technical Report). *Pure Appl. Chem.* **1995**, *67*, 2, 331–343.

(3) Horwitz, W. Harmonized Protocol for the Design and Interpretation of Collaborative Studies. *TrAC Trends Anal. Chem.* **1988**, *7*, 4, 118–120.

(4) Shapiro, S. S.; Wilk, M. B. An Analysis of Variance Test for Normality (Complete Samples). *Biometrika* **1965**, *52*, 3/4, 591.

(5) Yazici, B.; Yolacan, S. A Comparison of Various Tests of Normality. *J. Stat. Comput. Simul.* **2007**, *77*, 2, 175–183.

(6) Davies, P. L. Statistical Evaluation of Interlaboratory Tests. *Fresenius' Zeitschrift für Anal. Chemie* **1988**, *331*, 5, 513–519.

(7) Otto, M. *Chemometrics: Statistics and Computer Application in Analytical Chemistry*; John Wiley & Sons, 2023.

(8) Doppelt, P.; Hallais, G.; Pinson, J.; Podvorica, F.; Verneyre, S. Surface Modification of Conducting Substrates. Existence of Azo Bonds in the Structure of Organic Layers Obtained from Diazonium Salts. *Chem. Mater.* **2007**, *19*, 18, 4570–4575.

(9) Greenwood, J.; Phan, T. H.; Fujita, Y.; Li, Z.; Ivasenko, O.; Vanderlinden, W.; Van Gorp, H.; Frederickx, W.; Lu, G.; Tahara, K.; Tobe, Y.; Uji-i, H.; Mertens, S. F. L.; De Feyter, S. Covalent Modification of Graphene and Graphite Using Diazonium Chemistry: Tunable Grafting and Nanomanipulation. *ACS Nano* **2015**, *9*, 5, 5520–5535.
